# Autonomous UAV System for Cleaning Insulators in Power Line Inspection and Maintenance

**DOI:** 10.3390/s21248488

**Published:** 2021-12-20

**Authors:** Ricardo Lopez Lopez, Manuel Jesus Batista Sanchez, Manuel Perez Jimenez, Begoña C. Arrue, Anibal Ollero

**Affiliations:** GRVC Robotics Laboratory, University of Seville, Avenida de los Descubrimientos, S/N, 41092 Seville, Spain; ricloplop2@us.es (R.L.L.); manbatsan@alum.us.es (M.J.B.S.); mpjimenez@us.es (M.P.J.); aollero@us.es (A.O.)

**Keywords:** UAVs, inspection and maintenance, mobile robots, insulators

## Abstract

The inspection and maintenance tasks of electrical installations are very demanding. Nowadays, insulator cleaning is carried out manually by operators using scaffolds, ropes, or even helicopters. However, these operations involve potential risks for humans and the electrical structure. The use of Unmanned Aerial Vehicles (UAV) to reduce the risk of these tasks is rising. This paper presents an UAV to autonomously clean insulators on power lines. First, an insulator detection and tracking algorithm has been implemented to control the UAV in operation. Second, a cleaning tool has been designed consisting of a pump, a tank, and an arm to direct the flow of cleaning liquid. Third, a vision system has been developed that is capable of detecting soiled areas using a semantic segmentation neuronal network, calculating the trajectory for cleaning in the image plane, and generating arm trajectories to efficiently clean the insulator. Fourth, an autonomous system has been developed to land on a charging pad to charge the batteries and potentially fill the tank with cleaning liquid. Finally, the autonomous system has been validated in a controlled outdoor environment.

## 1. Introduction

The inspection and maintenance of power lines represent a significant economic cost for electricity supply companies. These tasks need to be performed periodically and can result in failures, leading to economic and material losses.

The power transmission system often has to cover long distances, and it has routes that are difficult to access by land.

Moreover, maintenance tasks are performed at high altitudes and require the use of helicopters, ropes, and elevating platforms for tasks such as the installation of bird flight diverters or cleaning power line devices. Particularly, power line insulators are cleaned while the line is energized.

An electrical flash-over may occur through the air between the tower and the conductor, causing blackouts, brownouts, and damage to the installation if the insulators are contaminated. The aim of cleaning it is to remove oxidation and other deposits using a water jet.

Research in aerial robotics for industrial inspection is providing autonomous solutions to traditional methods. The performance of aerial robotics has been proven in fields such as civil engineering [1,2], agriculture [3], the mining industry [4], or conveying systems [5].

In addition, the use of UAVs for tasks that are not purely inspection has increased. Particularly, UAVs are already being used for agriculture monitoring and the spraying of crops [6].

Moreover, in [7], a low-cost fumigation system which has been redesigned and implemented in a UAV is shown. The results show that there is a big difference in application costs between using the system on UAVs or using a conventional hydro-pneumatic sprayer.

Visual inspection operations of power lines are of relevance due to the cost, complexity, and risk of human inspection caused by the need to cover large areas using manned helicopters.

Traditional monocular [8,9] and stereo [10] cameras have been implemented in aerial platforms to detect power line defects. For instance, landing algorithms on power lines have been developed and validated in real environments with an aerial system composed of a LiDAR and a monocular camera for inspection and maintenance [11]. Thermal cameras have been widely used to detect damaged components with an over-heating problem [12] including insulators [13,14]. The recent advances in the computational power of onboard computers have led to real-time applications on UAVs. It is common to find applications in which neural networks are used to detect or recognize elements to interact with them [15]. The detection of insulators has been addressed by different approaches. Due to the different aspect ratios and scales, a faster region-based convolutional neuronal network (R-CNN) model has been implemented [16]. Moreover, the detection of composite and porcelain insulators has been developed on cluttered backgrounds with a single shot multibox detector (SSD) [17].

Despite the considerable potential of UAVs for inspection and maintenance, flight autonomy has been a major constraint. A wide range of wireless charging systems has been developed [18]. A platform for UAVs consisting of two coils transmitting energy using an oscillating magnetic field [19] has been designed. In addition, a self-leveling platform for small UAVs that can be installed on any type of surface [20] has been implemented. Usually, vision-based control is needed to ensure the necessary precision to land on the charging pad [21].

The use of control algorithms for landing UAVs on platforms is well studied. Image-based visual servoing algorithms have been developed to control the UAV while tracking the platform [22]. Moreover, a model of predictive control for autonomous landing under wind disturbances on a moving platform [23] has been developed.

The main contribution of this paper is the design and experimental validation of a fully autonomous application for cleaning insulators on power lines. First, a lightweight cleaning tool that can be installed on a UAV has been designed. Second, the soiled areas of the insulator have been segmented, and an algorithm has been developed to obtain a sequence of optimal points to clean the insulator. Finally, an algorithm has been developed that estimates the fluid trajectory to hit the soiled areas.

The remainder of the paper is organized as follows. Section 2 describes the application and how the cleaning tool was designed and developed, the insulator detection and soil segmentation, and the descent algorithm to land on the charging pad. Section 3 shows the experimental validation of the system in an outdoor environment. Finally, Section 4 outlines the conclusions of this work.

## 2. System Description

**Figure 1:**
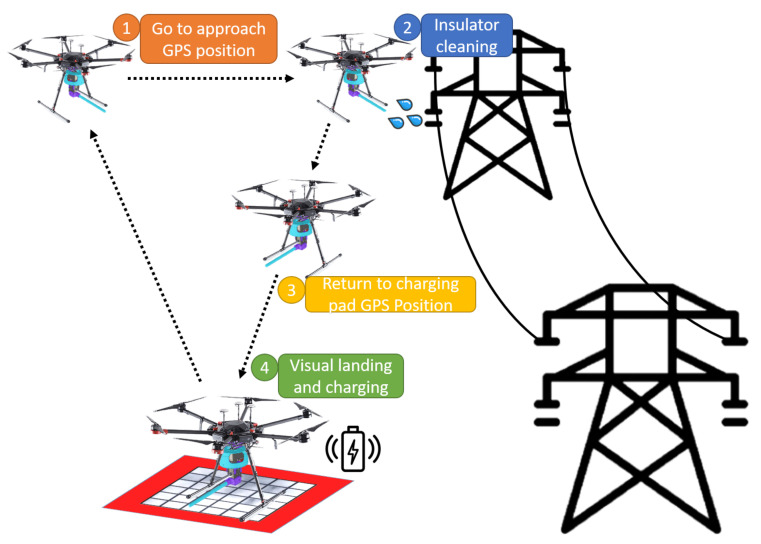
Conceptual design of the operation. (**1**) The UAV is sent to the GPS position of an insulator. (**2**) Visual local control is performed while locating and cleaning the areas that need the maintenance of the insulator. (**3**) When the operation is finished or batteries need to be recharged, the UAV returns to the GPS position of a charging station. (**4**) When it reaches the position, an autonomous vision-based descent is performed.

**Figure 2:**
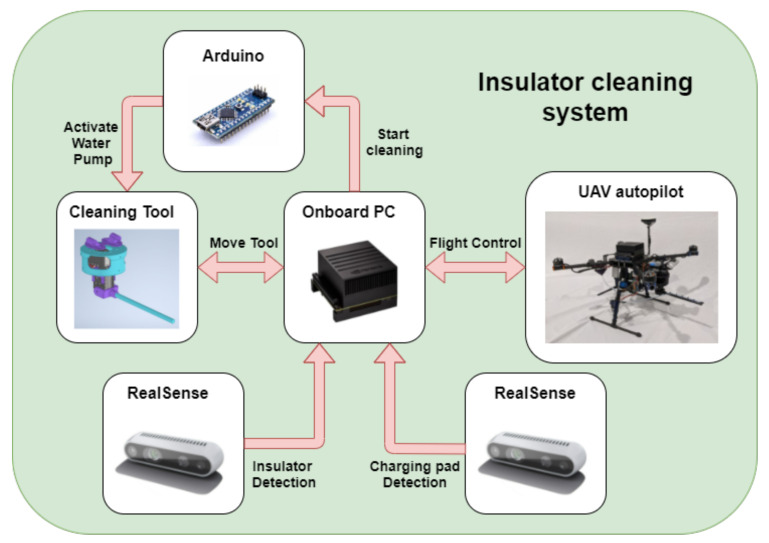
Conceptual scheme of the systems involved in the application.

The main goal of this work is to develop an autonomous UAV system for insulator cleaning in power lines as shown in Figure 1. First, the system uses a global positioning system (GPS) point to approach the target area. Then, a vision algorithm using a convolutional neuronal network (CNN) provides the location of the insulator to control the UAV. The cleaning tool aims and shoots a water jet on a point sequence given by an algorithm with a semantic segmentation neuronal network that detects the soiled areas of the insulator. The maximum payload that the UAV can carry will be mostly used for storing the cleaning liquid. For that reason, the tool developed for cleaning the insulators has been designed to minimize weight. When the battery or liquid tank level is low, the system returns to the charging pad. Finally, a vision-based algorithm for autonomous landing on a charging pad has been developed. The overall system structure can be seen in Figure 2.

### 2.1. Insulator Cleaning Tool

This section describes the custom tool used to clean the insulators. It has been designed to be small and lightweight, so it can be embedded even in small aerial robots.

The cleaning tool has been built with two Dynamixel AX-12A servomotors that provide the necessary degrees of freedom (DOFs) and a good power-to-weight ratio. The purpose of these DOFs is to allow the tool to aim at the insulator while flying, compensating for the oscillations and disturbances that the UAV might suffer.

Meanwhile, the cleaning liquid is driven by a water pump through a flexible rubber tube to the nozzle.

A micro board and a relay have been implemented to activate the water pump to control the flow of the liquid. Given the capacity of the tank, 0.44 L, and the flow rate of the water pump, 240 L/H, the algorithm estimates when the tank is going to be empty and activates a signal to decide whether to return to the charging pad or not.

**Figure 3:**
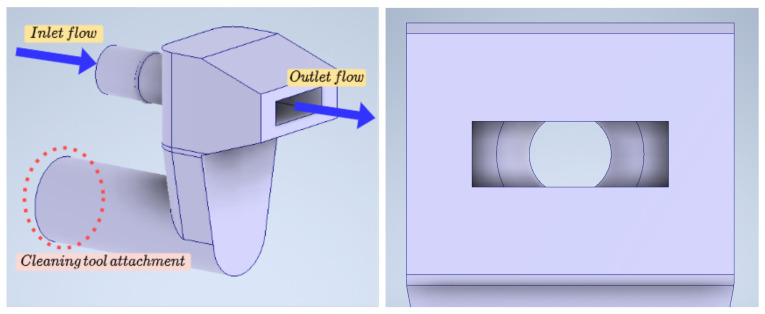
Nozzle with variable cross-section design to increase range and dispersion.

In addition, a custom nozzle has been designed to increase the range and horizontal jet dispersion as shown in Figure 3.

The tool has been printed in polylactic acid (PLA). It is designed to aim downwards to prevent droplets of water from reaching critical systems such as propellers or the electronics.

**Figure 4:**
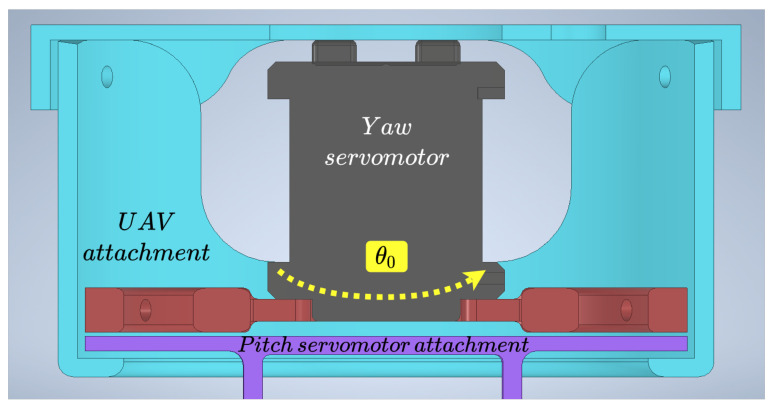
Design of the two-DOF cleaning tool.

One of the main elements to prevent a breakage of the yaw servomotor and maintain its performance is the red part in Figure 4, which holds the servomotor. The movement is transmitted to the purple piece, and as can be seen, if there were torsional moments, the blue piece that surrounds the previous ones would prevent the possible breakage of the servomotor. The purple piece at the bottom is the one that holds the cleaning tool to the UAV. This system has four silicone dampers, which connect the UAV body with the pointing tool, to avoid excessive vibrations between the UAV and the tool.

**Figure 5:**
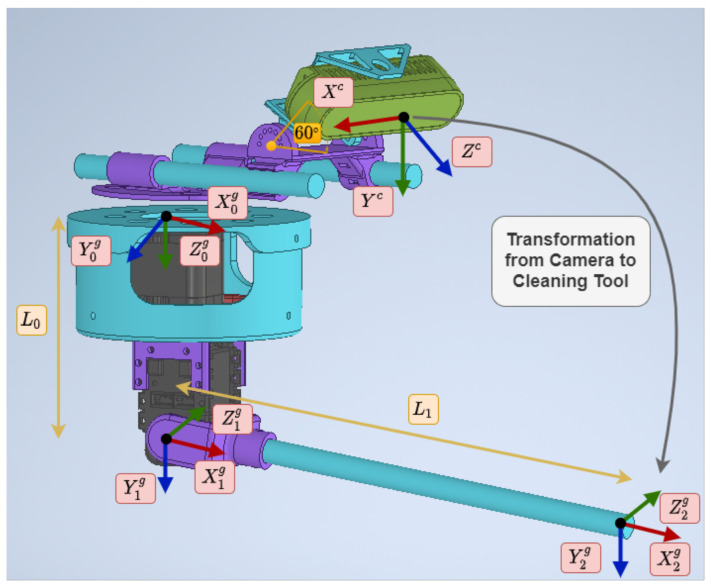
Schematic used to calculate the kinematics of the system along with the reference axes employed.

To move the described system, an analysis of its kinematics was carried out. The direct (1) and inverse (2) kinematics were obtained using the Denavit–Hartenberg method [24,25]. The scheme used can be seen in Figure 5, and the parameters are shown in Table 1. These parameters were applied to obtain the direct kinematics of the tool, which is used to know the transformation from the camera to the end-effector. In the equations, $\theta_{0}$, and $\theta_{1}$ are the joints that control the yaw and pitch, and $P=(X_{0}^{e},Y_{0}^{e},Z_{0}^{e})$ is the position of the end-effector from the tool coordinate frame.

(1) $$\begin{array}{c} X_{0}^{e}=L_{1}\cos\theta_{0}\cos\theta_{1}\\ Y_{0}^{e}=L_{1}\cos\theta_{1}\sin\theta_{0}\\ Z_{0}^{e}=L_{0}+L_{1}\sin\theta_{1} \end{array}$$

(2) $$\begin{array}{c} \theta_{0}=\arctan\frac{Y_{0}^{e}}{X_{0}^{e}}\\ \theta_{1}=\arcsin\frac{Z_{0}^{e}-L_{0}}{L_{1}} \end{array}$$

### 2.2. Targeting System

The targeting system has two goals: to evaluate whether the target point for cleaning can be reached and to minimize the distance between the fluid trajectory and the target point.

The flow trajectory is determined by the velocity that the pump is capable of transmitting to the fluid at the nozzle outlet. Therefore, the fluid outlet parameters have been estimated experimentally to choose a mathematical model. Due to the low velocity of the fluid, the air resistance can be neglected. Therefore, it has been determined that the parabolic trajectory can be accurate for the range where the UAV is at least 1.5 m away from the insulator [26,27]. The deviation from the estimated trajectory is mostly caused by the wind.

**Figure 6:**
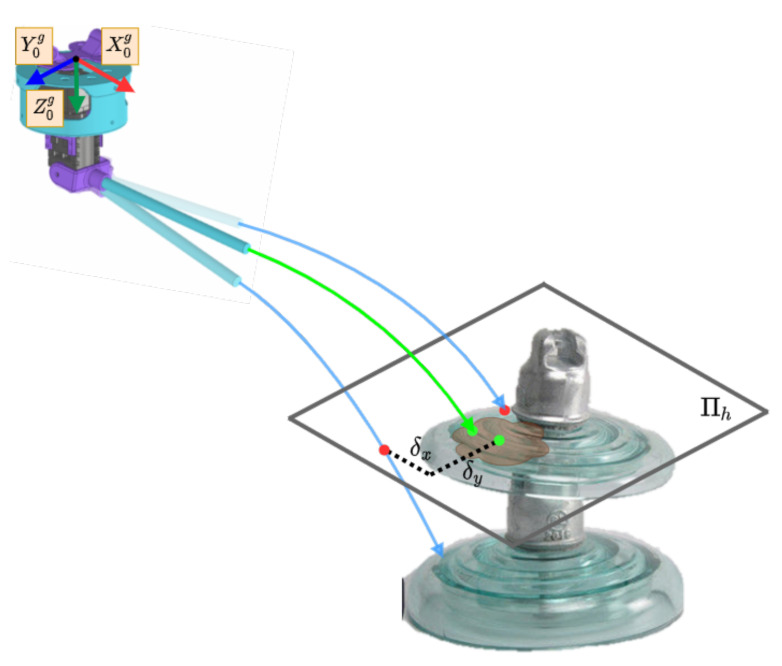
Diagram of the targeting system for choosing the optimal joint variables to hit the target point of the insulator.

The algorithm starts when the system detects a point to clean that is less than 1.5 m from the UAV. First, the target point is transformed to the reference frame of the cleaning tool. Second, the joints $\theta_{0}$ and $\theta_{1}$ are defined to evaluate the best configuration. Third, using direct kinematics, the point of the end-effector is obtained using direct kinematics. Then, the parabolic trajectory of the fluid is calculated for that configuration. A point is obtained from the intersection of the trajectory in the horizontal plane ($\Pi_{h}$) with the target point. This algorithm attempts to minimize the distance $d=\sqrt{\delta_{x}^{2}+\delta_{y}^{2}}$, as shown in Figure 6. Finally, when the entire workspace has been covered, the joints are sent to the cleaning tool. Algorithm 1 summarizes the process.
**Algorithm 1** Algorithm to obtain the optimal joints to hit the target.1:$TargetPoint^{camera}\leftarrow CleaningZoneDetection\left(RGB,Deph\right)$2:$TargetPoint^{tool}\leftarrow TransformToTool\left(TargetPoint^{camera}\right)$3:MinDistance←0.5m4:**for**$\theta_{0}^{i}=Min\left(\theta_{0}\right)$ to $Max(\theta_{0})$ **do**5:  **for** $\theta_{1}^{j}=Min\left(\theta_{1}\right)$ to $Max(\theta_{1})$ **do**6:    $Joints\leftarrow (\theta_{0}^{i},\theta_{1}^{j})$7:    WorkSpacePoint←DirectKinematic(Joints)8:    $Point^{tool}\leftarrow FluidTrajectory\left(WorkSpacePoint\right)$9:    $Distance\leftarrow (TargetPoint^{tool},Point^{tool})$10:    **if** Distance<MinDistance **then**11:       MinDistance←Distance12:       FinalJoints←Joints

### 2.3. Insulator Detection

The cleaning task requires the UAV to be positioned close enough to the insulator for the cleaning liquid jet to reach the desired point. For this purpose, a vision algorithm to detect and track the insulator has been developed. The detection has been implemented by a CNN. Then, a NVIDIA Jetson Xavier AGX was chosen, which allows the detection to be performed with a low inference time.

To locate the insulator, a positioning system has been developed. First, the YOLO v4 Tiny neural network [28] is used to detect the device in the image. The CNN has been trained using open datasets and around 4000 additional images of insulators that have been collected in several environments with the aerial platform.

This detection system is executed in real-time on the UAV’s onboard computer. To improve the detection rate, an implementation with TensorRT has been used, providing a higher frequency of the bounding box, which improves the control loop. Table 2 shows a comparison between the different implementations on a validation dataset.

Once the device is detected, the resulting bounding box is used to feed a lightweight Kalman tracker [29].

**Figure 7:**
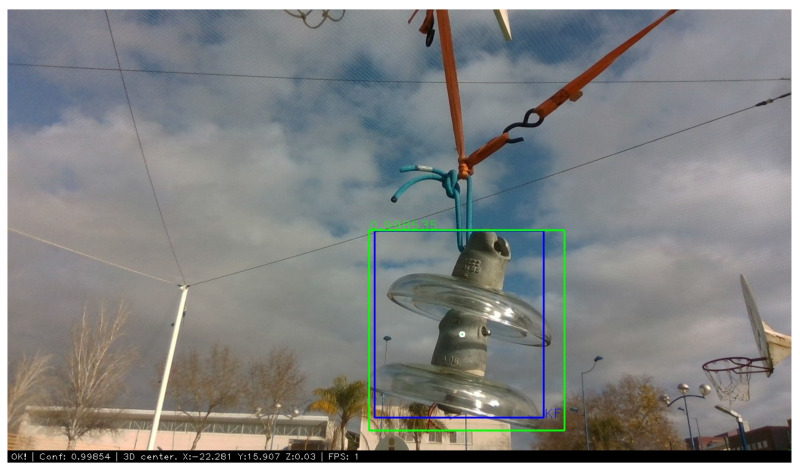
Insulator detection using YOLO v4 Tiny with TensorRT implementation (green bounding box) and tracker result (blue bounding box).

This tracker ensures continuity when the detection is lost between a few frames of real detection, and it reduces the effect of outliers. Figure 7 shows the bounding box of the implemented Kalman tracker in blue and the detection provided by the YOLO v4 Tiny network in green.

Then, a foreground extraction algorithm has been applied on the bounding box to segment the insulator and to obtain its centroid in the depth image. RGB and depth images have been aligned using CUDA, which reduces the processing time.

Finally, to improve the continuity of the detected position, another Kalman filter is implemented. This filter provides a smoother estimation of the 3D position of the device and reduces the noise effect introduced by the depth estimation of the camera.

### 2.4. Cleaning Zone Detection

Once the UAV maintains its position close enough to the insulator, a segmentation network is used to differentiate the areas that need to be cleaned from the ones that are already clean.

The semantic segmentation network used is based on a fully convolutional network FCN-ResNet101 and has been implemented using the Nvidia inference library for Jetson that allows the use of TensorRT and FP16 precision to decrease the inference time. A dataset has been created with about 2000 images of the insulator in various states of soiling that have been manually labeled. The network has been trained with 80% of the images and validated with the remaining 20%.

**Figure 8:**
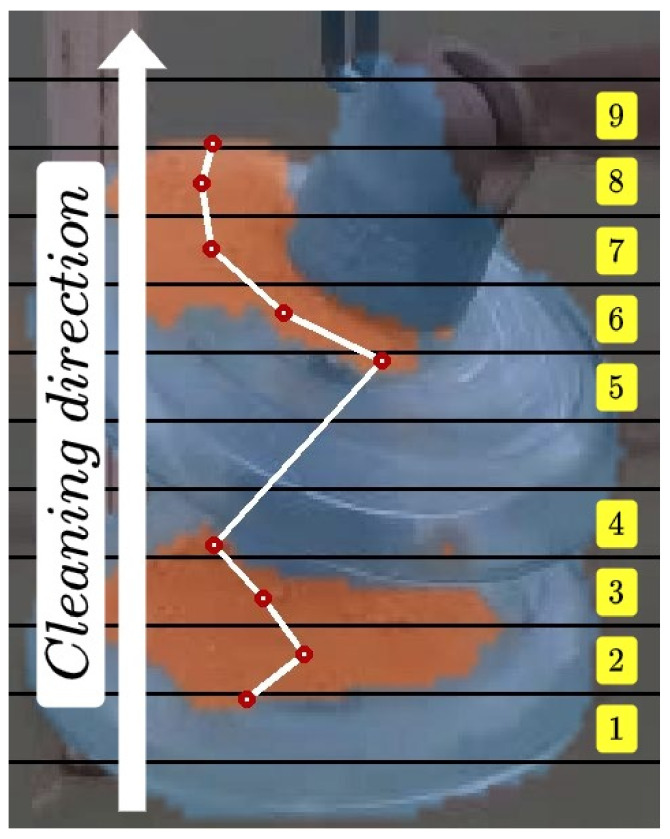
Soiled area segmentation and cleaning trajectory generated by the algorithm.

Using the segmentation mask obtained from the network, a system that defines the path to follow by the cleaning tool was developed. To generate this path, the image resulting from the segmentation of the soiled areas has been divided into equal horizontal segments as can be seen in Figure 8. The centroids of these divisions are used alongside the aligned depth image to obtain the points to be followed by the tool. This algorithm is performed online at a rate of 40 ms, so these points and the path calculated dynamically change during the cleaning operation. The points delivered to the cleaning tool are fed from the bottom up, as the cleaning of electrical equipment is carried out in this way.

### 2.5. Autonomous Landing on Charging Pad

**Figure 9:**
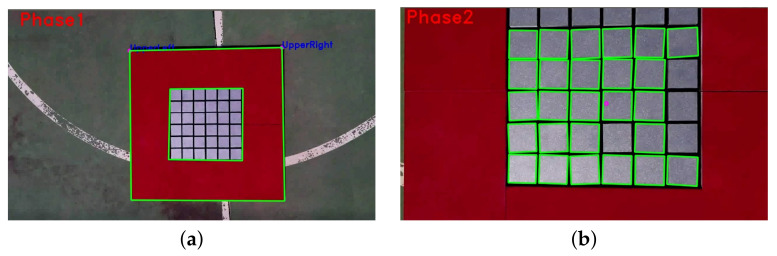
Two-phase detection algorithm. (**a**) Phase 1; (**b**) Phase 2.

The application relies on the ability of the UAV to charge itself, since cleaning multiple insulators would drain the battery. Therefore, a platform that integrates a commercial charging pad [30] has been designed and built. The platform is designed to be clearly visible from a high altitude to facilitate landing, as shown in Figure 9a.

The descent maneuver is performed using a position-based visual servoing (PBVS) algorithm [31]. Due to the low velocity during the descent maneuver, it has been assumed that the image plane is parallel to the ground and the depth has been estimated using the depth image provided by the camera.

The detection and tracking of the platform are essential to make a stable and reliable landing maneuver. In many cases, the platform will be located at a distance of more than 7 m from the UAV. A lightweight algorithm is required to make it feasible to maneuver the vehicle even in adverse wind conditions that require responsive control. The detection of the charging pad was performed using an algorithm with two phases.

During the first phase, the camera sees the target partially or completely, at a minimum distance of two meters. Color segmentation [32] and shape matching were performed using HSV (hue, saturation, value) thresholding to detect the outside contour. To be able to start charging, the landing gear must be within the inner boundary of the platform. It is necessary to estimate the relative yaw difference between the platform and the UAV. Due to the small available area, a safe landing can only be achieved if the yaw difference is less than 15º and the distance to the center is less than 15 cm.

In the second phase, the algorithm tracks the platform center and yaw misalignment in close range. The system detects multiple inner squares using the HSV threshold and the Hough transform. Since the cells of the charging platform are highly reflective, an online algorithm has been developed to maximize the number of squares it detects by preprocessing the image by applying convolutional filters with different kernel sizes and changing HSV thresholds. The two phases can be seen in Figure 9.

**Figure 10:**
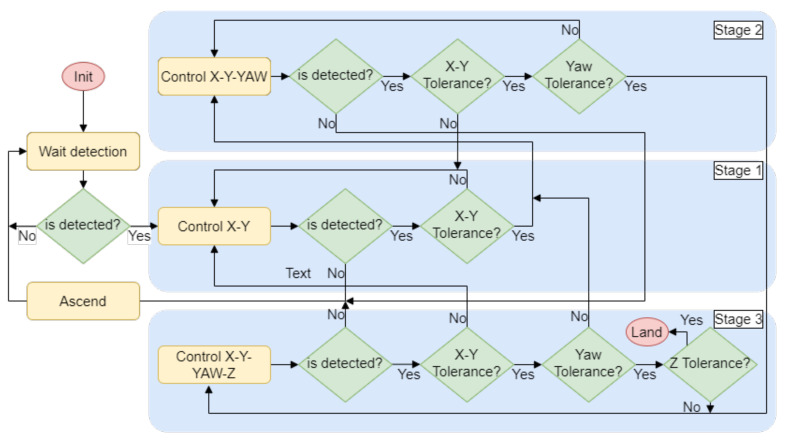
State machine used for the descent and landing maneuver.

A landing state machine has been developed to control the UAV through the descent. When the system needs to return to the charging pad, the state machine is started along with the first detection phase. The algorithm consists of three stages as can be seen in Figure 10.

The first stage detects the landing station for the first time and controls the UAV to center the target in the X–Y plane. When the UAV has succeeded in reducing the distance to the center of the platform by a threshold, it continues to the next stage. The second is used to align the UAV with the platform by adding to the control a yaw rotation. The third stage initiates when the X–Y distance and yaw rotation thresholds are reached. Then, the descent maneuver commences while keeping the platform centered and aligned, controlling four DOFs. If any of the conditions are not fulfilled, the system will return to the previous stage until they are satisfied. When the distance to the platform is less than 50 cm on the Z-axis, the landing is performed. The limits set for switching between stages are dynamically calculated depending on the height of the UAV.

## 3. Experimental Validation

This section shows the experimental results and analyzes each subsystem that composes the application. The autonomous insulator cleaning system has been validated in a controlled outdoor environment. This work does not attempt to analyze the interaction of flight systems with electromagnetic interference generated in proximity [33] or in contact [34] with high-voltage power lines. Thus, an insulator has been placed in a controlled outdoor environment.

**Figure 11:**
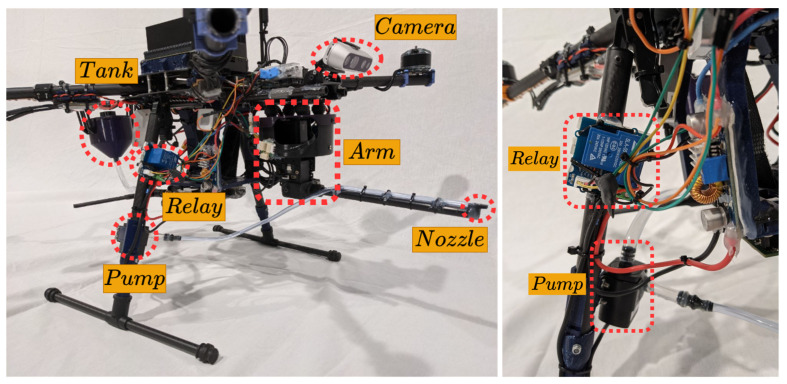
UAV hardware setup.

The multirotor used in this work has been developed specifically for this application. A T-motor Navigator MN4014 of 330 KV motor has been integrated into the Tarot XS690 frame. The autopilot implemented is the Pixhawk 2. The power supply consists of a LiPo 6 s 7000 mAh battery for the motors and a LiPo 4 s 3000 mAh battery that supplies the onboard computer, the arm, the Arduino, and the pump. The total weight of the platform is 4.5 kg plus 0.44 kg of cleaning liquid that the tank holds. This setup delivers a nominal thrust of 1.5–1.6 kg per axis with a maximum flight time of 7 min. Figure 11 shows the hardware setup of the UAV.

### 3.1. Visual Control

**Figure 12:**
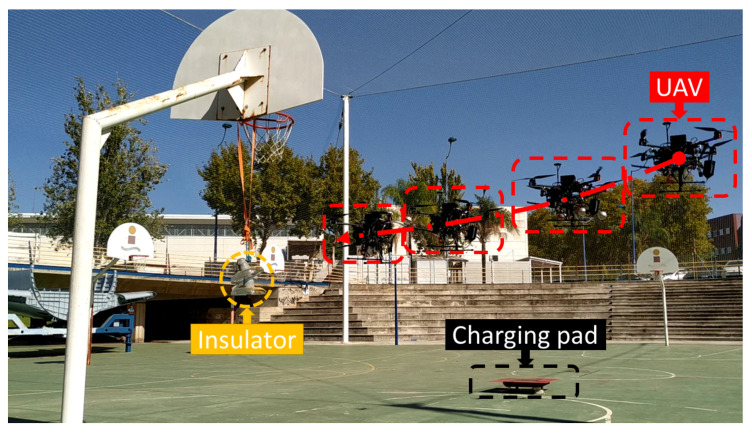
Path followed by the UAV.

The experiments have been performed in an outdoor environment using the Intel RealSense D435i camera as described in Section 2.3 at a resolution of 640 × 480 px. A mission has been conducted in which the UAV takes off from the landing platform. Then, the system performs an approaching maneuver to the insulator. At a distance where the insulator can be reached, the local control algorithm begins, as can be seen in Figure 12. The targeting system is reliant on the visual control performed by the multirotor; thus, the local control has to be precise and stable to achieve the proper cleaning of the insulator.

The visual guidance has been performed using a cascade control that applies a simple PID algorithm [35] for controlling the multirotor. The position controller is fed with the position of the insulator. To have a wider range of vision, the camera has been positioned with a 30º pitch rotation. Then, it has been estimated that the optimum distance for cleaning is 1.5 m on the Y-axis and −0.5 m on the Z-axis in an ENU (east–north–up) coordinate system.

**Figure 13:**
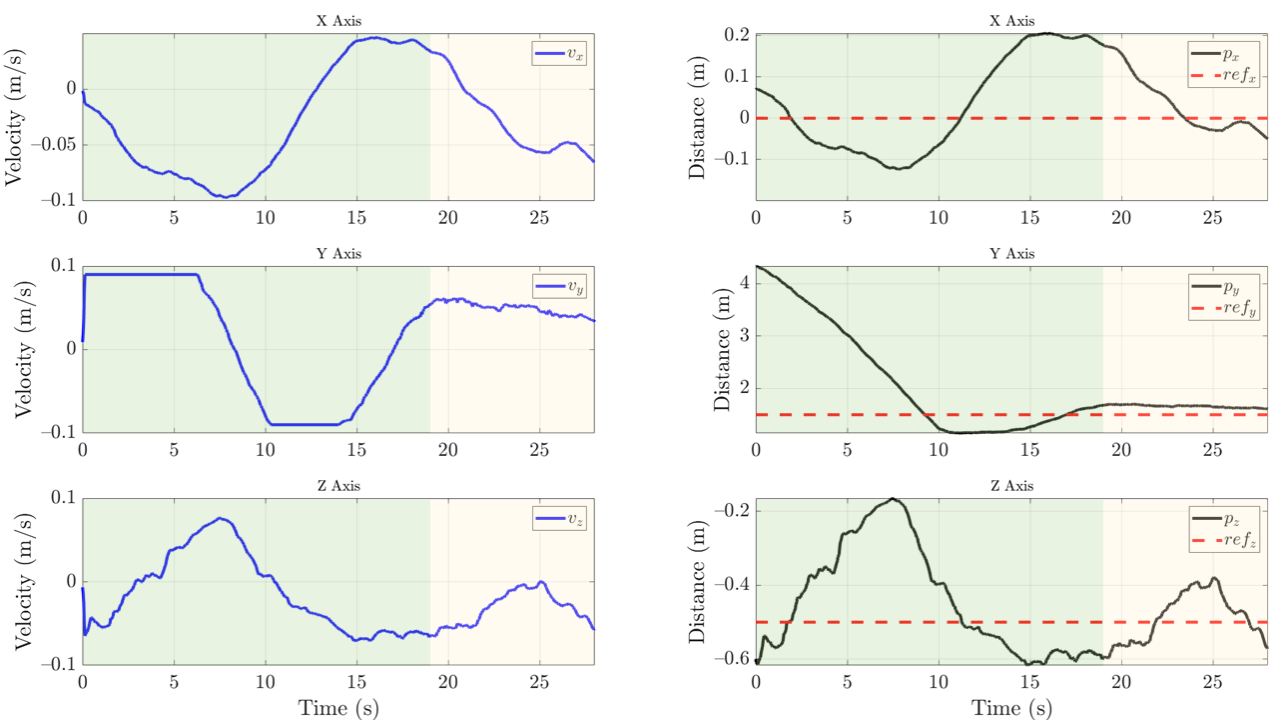
Visual control of the UAV with the control signal (**blue line**), position estimation of the insulator (**black line**), and reference (**dotted red line**). Once the insulator is detected, the UAV performs the approach phase indicated by the green area. When the UAV is stabilized on the three axes below a threshold for three seconds, the cleaning phase indicated by the yellow area begins.

The control signal and the position of the insulator can be seen in Figure 13. First, the insulator is detected at a distance of 4.5 m on the Y-axis and the UAV starts the approach maneuver, saturating the forward velocity. On the X-axis, an overshoot of at most 20 cm of error, which is within the reach of the cleaning tool, occurs. When the visual control manages to stabilize the UAV between an error of 20 cm on the X-axis, 30 cm on the Y-axis, and 10 cm on Z-axis for 3 s, the cleaning phase starts.

Then, during the cleaning phase, the targeting and cleaning system is capable of performing the task while the UAV compensates for the weight dumped from the cleaning liquid tank. The drift in the X-axis and Y-axis is less than 30 cm. Moreover, the insulator is always in sight, and the tracking that feeds the position control is stable.

Finally, the results show that the UAV can be controlled with the payload during the approach and cleaning phase.

### 3.2. Targeting System and Cleaning Insulator Results

The targeting and cleaning system starts when the UAV comes within range of the insulator. First, the relay is activated. Then, the arm is driven in pitch to prune the air bubbles in the liquid transmission system. Therefore, a sequence of calibration points is performed at the start-up. This makes the cleaning liquid jet start at maximum pressure so that it can reach the estimated point from the beginning. Second, the system described in Section 2.4 estimates the points to be reached by the water jet to clean the insulator. Third, the arm guided by the points of the vision system sequentially reaches the point and clears the soiled area. Finally, the cleaning task ends when it has been detected that there is no soil in the insulator or the tank cleaning liquid has been completely emptied.

**Figure 14:**
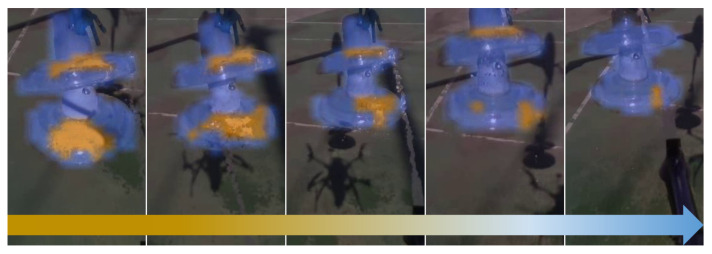
Evolution of soiled areas in the insulator.

Figure 14 shows the onboard camera view of the insulator during the cleaning process, where it can be better appreciated how the cleaning tool works.

The cleaning tool is able to sequentially reach the target point and decrease the soil. The graph shows the path followed by the tool and the path generated from the coordinates of the camera transformed to the reference system of the tool. The UAV is in constant motion during cleaning, so the joints are compensating such motion to impact the target. However, the inertia of the lines means that when it varies in *X*, *Y*, or *Z* directions, there is the same variation in the tool to compensate for this new movement towards the achievable position.

**Figure 15:**
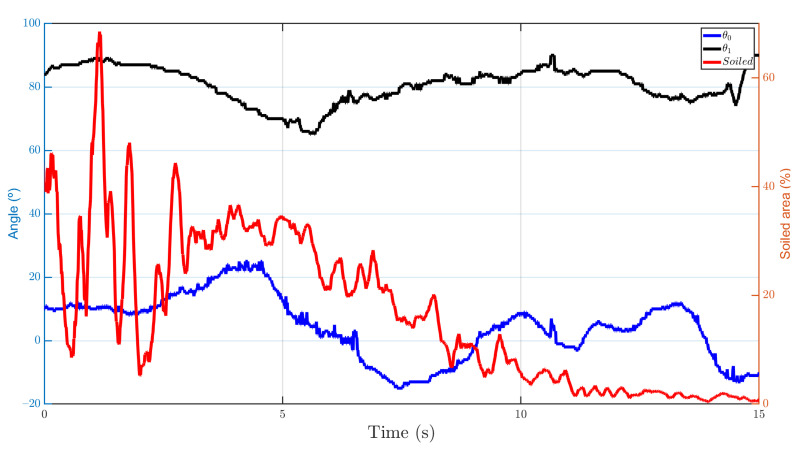
Joint variables and percentage of soiled area cleaned over time by the cleaning tool.

The percentage of the area that is cleaned has been measured using the area in pixels from the segmentation of the clean and soiled areas. As can be seen in Figure 15, about 90% of the soil is removed from the insulator. However, during the first three estimated points, the soil appears to be spread out. Although, once the next points are reached, this is significantly reduced as more cleaning liquid is applied to the surface.

It should be noted that the parabolic trajectory estimation has been essential to obtain the best configuration of the joints, since the drop of the water jet is significant due to the low pressure and velocity of the fluid at the nozzle outlet. Nearly 30 cleaning experiments have been carried out under different soiling states, of which an average of about 70% of the dirty area of the insulator has been reduced.

### 3.3. Landing System

After cleaning the insulator, the UAV moves to a safe position and returns near the charging station via GPS waypoints. The algorithm described in Section 2.5 is then activated, and the descent maneuver begins once the platform is detected. We chose to use a resolution of 640 × 480 px, since the Intel RealSense D435i camera can stream images at 60 Hz with this resolution. This configuration is optimal because the detection and tracking algorithm can estimate the position of the center of the platform in around 25 ms and therefore send the control signal at 40 Hz.

During the descent maneuver, the arm is placed in a symmetrical position to minimize the moments that can be generated by the weight distribution. In addition, the cleaning liquid tank is empty or nearly empty. Therefore, the descent control system is more precise due to inertia reduction. This is a key factor since the landing maneuver must be precise (around 20 cm in the horizontal plane) due to the dimensions of the platform, so control in the horizontal plane of the UAV becomes crucial.

**Figure 16:**
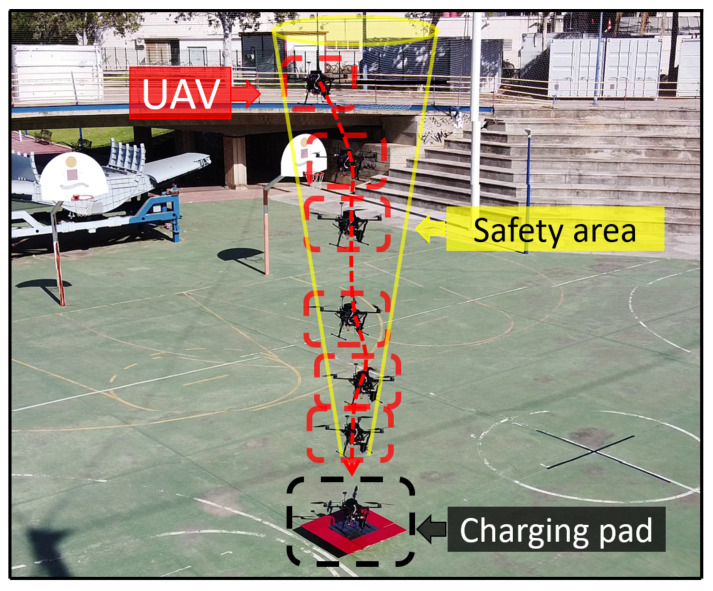
Trajectory followed by the UAV during landing—example of the cone made for a safer descent.

Figure 16 shows the trajectory followed by the UAV during the descent maneuver. The state machine and the safety cone that is applied ensure that the UAV cannot land if the safety conditions are not satisfied.

**Figure 17:**
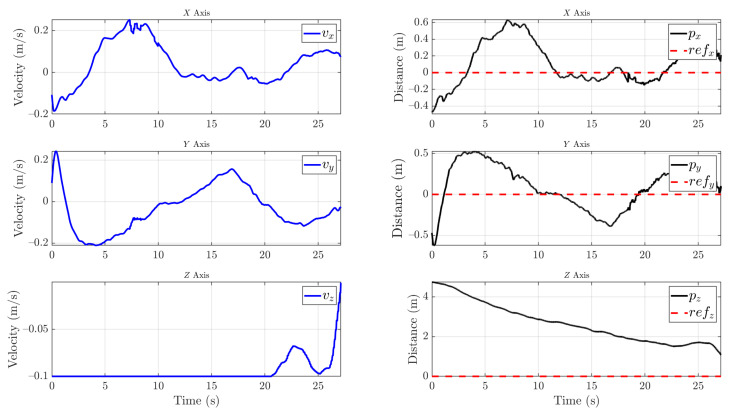
Control signals sent to the autopilot during landing (**blue line**) and charging pad position (**black line**) with the reference (**dotted red line**). The UAV aligns with the platform in the horizontal plane while descending at a constant speed. The second phase of detection begins at 1.8 m, slowing the descent speed and waiting for the optimum horizontal plane alignment to achieve a safe landing.

Nearly 30 descent maneuvers have been made. The visual descent control can be seen in Figure 17. The mean error obtained during the experiments in the X and Y axes is 12 cm from the center of the platform. This range is acceptable for the platform to charge since at least one leg must be within the cell. The results show that the landing is safe and robust. However, the UAV manages to land at an altitude of 7 m on average in 25 s. The autonomous cleaning operation video of an insulator can be seen in Supplementary Materials.

## 4. Conclusions and Future Work

The work presented in this article shows a completely autonomous cleaning system for power line insulators. This system offers an alternative to reduce risk exposure during power line maintenance. Additionally, the system can detect when it needs to recharge its batteries or cleaning liquid, return to the charging station, and land autonomously.

Due to the interference caused by power lines, this system offers more precise local positioning than GPS in the proposed environment. Given the ability of UAVs to move nimbly, a good position for cleaning the insulators can be obtained. The system is adaptable from small to larger aerial robots. Furthermore, any controller for UAVs that allows the vehicle to be moved using speed commands can be used.

Nevertheless, the application has to be validated with insulators on an energized power line. The system has been developed not to be dependent on GPS signals near the power line due to electromagnetic interference; therefore, it is necessary to measure the optimal range to start local control for cleaning. If a longer range is required, the UAV must be equipped with a higher resolution camera. In addition, the segmentation network has to be trained with more images of soiled insulators. Hence, the creation of a larger dataset is necessary.

Future work should focus on increasing the pump pressure to improve the cleaning. However, this would change the estimation of the fluid trajectory. By increasing the velocity at the nozzle outlet, the trajectory would no longer look like a parabola, becoming an ellipsoid that increasingly depends on air resistance. Therefore, it would be necessary to replace the method developed in this work with a more detailed study of the jet. It should be noted that the UAV must compensate this force. This disturbance can be predicted, which would increase the need for a model-based control that would combine the UAV model and the model of the force caused by the fluid outflow. In addition, fluid loss in the tank can be taken into consideration and added to the model. Moreover, it is necessary to carry out experiments with insulator cleaning liquids to validate the flow transmission and pump system.

The system has a margin for improvement. The system will refill the liquid autonomously by targeting an onshore reservoir, reversing the flow in the transmission system. The landing camera can be replaced by a higher resolution camera with a higher or equal streaming rate to increase the distance at which the platform is detected. The servo motors used in the targeting system will be replaced by more precise and smaller encoder motors. Finally, an application with a simple user interface will be developed to tune the cleaning, control, and charging parameters.

## Figures and Tables

**Table 1:** Denavit–Hartenberg parameters of the manipulator.

	$\theta_{i}$	$d_{i}$	$\alpha_{i}$	$a_{i}$
Link 1	$\theta_{0}$	$L_{0}$	90°	0
Link 2	$\theta_{1}$	0	0	$L_{1}$

**Table 2:** Inference time between different object detector models.

	Inference Time (ms)
YOLO v4 Tiny	29.4
YOLO v4 Tiny + TensorRT	20.4

## Data Availability

Not applicable.

## Supplementary Materials

The following are available online at https://www.mdpi.com/article/10.3390/s21248488/s1, Video S1: Autonomous UAV System for Cleaning Insulators in Power Line Inspection and Maintenance.

## Abbreviations

The following abbreviations are used in this manuscript:

UAV	Unmanned aerial vehicle
DOF	Degrees of freedom
R-CNN	Region-based convolutional neuronal network
SSD	Single shot multibox detector
GPS	Global positioning system
CNN	Convolutional neuronal network
PLA	Polylactic acid
CAD	Computer-aided design
CNN	Convolutional neuronal network
PBVS	Position-based visual servoing
HSV	Hue, saturation, value
ENU	East–north–up
